# A Texture Based Pattern Recognition Approach to Distinguish Melanoma from Non-Melanoma Cells in Histopathological Tissue Microarray Sections

**DOI:** 10.1371/journal.pone.0062070

**Published:** 2013-05-17

**Authors:** Elton Rexhepaj, Margrét Agnarsdóttir, Julia Bergman, Per-Henrik Edqvist, Michael Bergqvist, Mathias Uhlén, William M. Gallagher, Emma Lundberg, Fredrik Ponten

**Affiliations:** 1 Department of Genetics and Pathology and Science for Life Laboratory, Rudbeck Laboratory, Uppsala University, Uppsala, Sweden; 2 Section of Oncology, Department of Oncology, Radiology and Clinical Immunology, Rudbeck Laboratory, Uppsala University, Uppsala, Sweden; 3 Science for Life Laboratory, KTH – Royal Institute of Technology, Stockholm, Sweden; 4 UCD School of Biomolecular and Biomedical Science, UCD Conway Institute, Dublin, Ireland; 5 OncoMark Limited, NovaUCD, Belfield Innovation Park, Dublin, Ireland; Ludwig-Maximilian-University, Germany

## Abstract

**Aims:**

Immunohistochemistry is a routine practice in clinical cancer diagnostics and also an established technology for tissue-based research regarding biomarker discovery efforts. Tedious manual assessment of immunohistochemically stained tissue needs to be fully automated to take full advantage of the potential for high throughput analyses enabled by tissue microarrays and digital pathology. Such automated tools also need to be reproducible for different experimental conditions and biomarker targets. In this study we present a novel supervised melanoma specific pattern recognition approach that is fully automated and quantitative.

**Methods and Results:**

Melanoma samples were immunostained for the melanocyte specific target, Melan-A. Images representing immunostained melanoma tissue were then digitally processed to segment regions of interest, highlighting Melan-A positive and negative areas. Color deconvolution was applied to each region of interest to separate the channel containing the immunohistochemistry signal from the hematoxylin counterstaining channel. A support vector machine melanoma classification model was learned from a discovery melanoma patient cohort (n = 264) and subsequently validated on an independent cohort of melanoma patient tissue sample images (n = 157).

**Conclusion:**

Here we propose a novel method that takes advantage of utilizing an immuhistochemical marker highlighting melanocytes to fully automate the learning of a general melanoma cell classification model. The presented method can be applied on any protein of interest and thus provides a tool for quantification of immunohistochemistry-based protein expression in melanoma.

## Introduction

Antibody-based proteomics provides an advantageous strategy for biomarker discovery efforts [1]. Immunohistochemistry (IHC) is a well established and accepted assay for labeling a specific protein in tissue, provided the availability of validated antibodies towards the target of interest. IHC assays use an antibody to specifically couple a candidate protein and a dye that makes the immunoreaction visible to the human eye in a microscope or a digital glass slide scanner. In routine IHC, hematoxylin a natural dye binding to nuclei acid, is added to the tissue specimen to highlight the cell nuclei in dark blue, cell cytoplasm and extra cellular matrix in light blue. The most common immunostaining combination, achieving the highest contrast target signal to background ratio, is diaminobenzidine (DAB) for visualizing the IHC signal with hematoxylin counterstaining. With the introduction of tissue microarray (TMAs) technology [2] a large number of tissue specimens can be analyzed simultaneously using IHC. Over the past decade, TMAs have become an established and crucial component of the cancer biomarker discovery and validation pipeline.

The Human Protein Atlas (HPA) project has been set up to allow for a systematic exploration of the human proteome using antibody-based proteomics [3], [4]. This is accomplished by combining, high-throughput generation of affinity-purified antibodies with protein profiling in a multitude of tissues and cells assembled in tissue microarrays. The Human Protein Atlas portal (www.proteinatlas.org) is a publicly available database [5] with over 10 million high-resolution images showing the spatial distribution and relative abundance of proteins in normal human tissues and a multitude of cancers, including 12 cases of malignant melanoma. IHC stained TMAs containing normal and cancer tissue are scanned to obtain the high-resolution images that are used for manual assessment of immunohistochemical outcome performed by pathologists. To leverage the true high throughput potential of antibody-based protein profiling in tissues, there is a need for fully automated scoring of IHC stained TMAs. Cancer tissue is complex and typically represents a pool of different cell types, including heterogeneous tumor cells and various normal cell types. For cutaneous melanoma, normal skin structures such as keratinocytes, blood vessels, inflammatory cells and fibroblasts in the dermal connective tissue are usually present together with cells and structures induced by the tumor, i.e. tumor stroma. For melanoma biomarker research applications where protein expression patterns in melanoma cells are analyzed, automated tools that can delineate melanoma cells from non-neoplastic cells with high accuracy would be of great benefit.

With the fast-paced changes in computing technology and recent advances on development of fast and high quality glass slide digitalization devices, an impact has also been seen in the field of histopathology [6]–[9]. Automated image analysis and pattern recognition approaches based on bright field microcopy have shown to be valuable to both clinical pathologists as well as to a broad range of researchers for analyzing high-throughput histopathological data [10]–[12]. The objectives of such automated image analysis based approaches is to analyze data efficiently, accurately, and reproducibly in high-throughput assays. As an alternative to the brightfield-based IHC system, fluorescence-based immunolabeled approaches such as AQUA have been used in both research and clinical settings [13]. The AQUA system is an established assay, combining a modified immunofluorescence protocol with image analysis to automate biomarker quantification in histopathological samples [14]–[16]. Although several studies have validated these fluorescence-based approaches, bright field IHC remains the standard assay used by pathologists. For both brightfield and fluorescence-based microscopy data, appropriate image processing and machine-learning techniques must be employed so as to extract, from images, as much relevant information as possible.

Cell nuclei object identification (also called segmentation) is a challenging step in medical image analysis and more particularly in digital pathology due to the resolution of the imaging data. One of the requirements of such crucial step is the accuracy and specificity of object identification. More particularly in pathology, image analysis cell segmentation defines the accuracy of the resulting cell texture and morphological measurements (i.e. features). In a second step, a process known as feature selection can identify the most useful information from the data, and reduce the dimensionality in such a way that the selected features represent the most significant aspects of the data. To improve the efficiency of classification algorithms, feature selection is used to identify and remove as much of the irrelevant and redundant information as possible. During this step, redundant features, such as features with low variability or not relevant for discriminating between the two cell nucleus population, are filtered out. Methods have been proposed for the segmentation of objects and feature extraction from microscopy-based images for various microscopy acquired data types, including histopathology [17], [18]. Several of these methods have been implemented in commercial image analysis frameworks such as Matlab (Mathworks) and Image-Pro (MediaCybernetics) and open source available tools such as CellProfiler [19] and ImageJ [20]. Segmentation and classification methods can be calibrated and adjusted for analysis of a broad range of microscopy image data including digital pathology data [21].

Here, we present a new method to fully automate the analysis of IHC-assessed protein expression in melanoma. An IHC marker was used to highlight melanoma cells in a tissue context. Image analysis was used to extract a set of morphological and texture features to characterize each type of cell and features distinguishing melanoma and non-melanoma cells were identified. Finally we present a classifier model applicable for IHC biomarker assessment in TMAs, that with high accuracy can distinguish between melanoma cells and non-melanoma cells. To our knowledge this is the first description of a method using a molecular marker for labeling both the training and testing set to fully automate this process.

## Methods

### Ethics statement

Ethical approval was given by the Central Ethical Review board of the Uppsala region (Regionala etikprövningsnämnden i Uppsala). Written consent was given by the review board for the use of human tissue from the discovery cohort (2005:U230) and the validation cohort (2005/U232) for melanoma research. The patients included in both studies signed an informed consent.

### Sample preparation and immunohistochemistry

The study focused on two independent cohorts of patients with diagnosed cutaneous melanoma. An initial discovery cohort was used to build a pattern recognition model for melanoma nuclear texture patterns. Samples included in the discovery TMAs were collected from a cohort of 264 patients diagnosed with malignant melanoma during the period 1993–2003. A validation cohort of 157 melanoma patients, diagnosed during the period 1982–2004, was also used in this study, for which tissue samples from primary or lymph node and metastatic tumor (if available) were collected [22]. TMAs, using 0.6 mm cylinders, were employed arraying up to 6 tissue core samples per patient (i.e. validation cohort). Commercially available antibodies were used as primary antibodies for IHC staining of Melan-A (Novocastra) and Ki67 (Dako). Automated IHC was performed as previously described [23], using 3,3′-diaminobenzidine (DAB) as chromogen to visualize antibody-target protein binding in tissue as a brown-black color signal.

### Manual scoring of IHC staining

Sections from the TMA representing the validation cohort were immunostained with the proliferation marker Ki67 to validate the pattern recognition model on IHC stained sections. Melanoma cells were manually counted and scored as positive or negative for Ki67 expression and an overall continuous ratio value of IHC positive cell over the total number of melanoma cells per tissue core was generated. This value was then compared to the automated reading of the melanoma cell pattern recognition and Ki67 quantification.

### Image acquisition

Digital images from immunostained TMA sections were acquired using an Aperio XT digital glass slide scanner (Aperio Technologies, Vista, California, USA) at 0.20 μm resolution. TMA digital slide images were further separated into individual tissue core images using the commercial software TMALab2 (Aperio Technologies). Extracted tissue core images were saved using JPEG2000 compression image format (Compression factor 1∶100).

### Quantification of IHC automated variation due to variability in staining and image acquisition settings

As previously described, inter and intra laboratory variability of both immunostaining and counterstaining are the primary cause of independent validation failure for most automated digital pathology applications [24]–[26]. To test for batch staining and counterstaining effect on the segmentation of cell nuclei from the Melan-A mask in the hematoxylin channel (i.e. H-CHANNEL), we included two different sets of TMAs (from discovery and validation patient cohorts) that had been stained and digitalized independently in the same laboratory. The respective staining intensity values were then compared among different batches.

### Image analysis and cell feature extraction

Image analysis and feature extraction were performed on IHC images using the open-source software CellProfiler R10997 [27] (http://www.cellprofiler.org) and Matlab R2011a (MathWorks, Apple Hill Drive, MA, USA).

**Figure 1:**
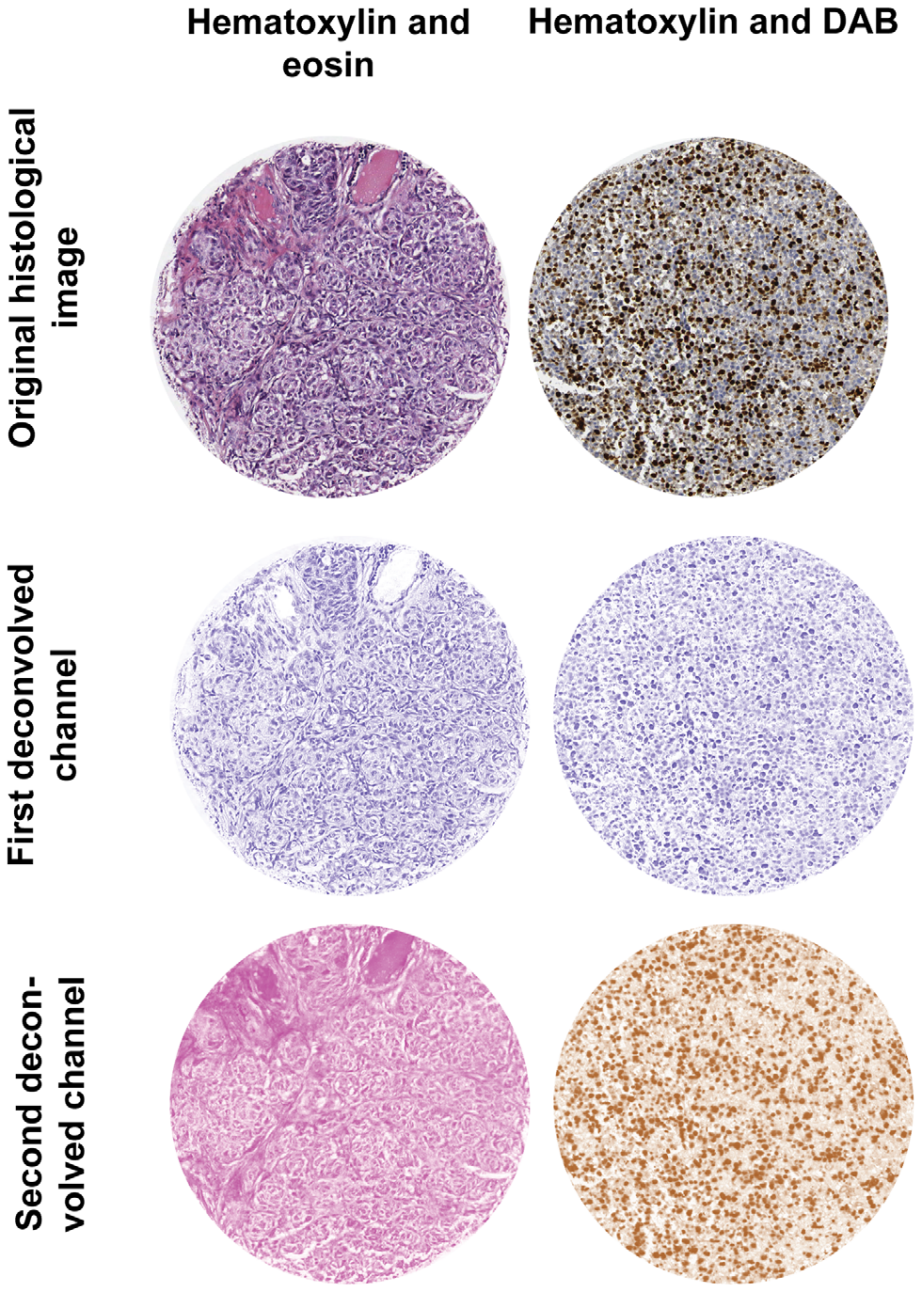
Dye separation. Using the color deconvolution plugin, an image of a tissue cores double stained with a counter staining dye (e.g. hematoxylin) and a second dye binding to cellular feature of interest (e.g. eosin or dye couple using IHC to a protein of interest) can be separated into two images highlighting amount and patterns of the feature of interest. The post deconvolution images are false-color coded in such a way that the RGB values of each pixels are associated to each dye absorption and according to the color properties of each original dye (i.e. pink for eosin, brown for DAB and blue for hematoxylin).

- **Dye separation.** Although the dyes used for labeling have different colors, namely brown for diaminobenzidine (DAB) and blue for hematoxylin, there exists complex overlapping spectra. To examine the photometric, morphometric and texture features of melanocyte nuclei, the relative contribution of each of the dyes to the resulting absorption spectrum needs to be separated. To estimate each dye contribution we used a color transformation technique, based on the original red-green-blue band information. The method is based on orthonormal transformations of the original RGB image depending on user-determined color information about the two stains [28]. After the color deconvolution, the images of each dye, namely H-CHANNEL (i.e. hematoxylin) and DAB-CHANNEL (i.e. diaminobenzidine) were used for further densitometry or texture analysis (Figure 1).
- **Melan-A mask generation.** A CellProfiler ruleset was developed to automatically extract Melan-A regions of interest (ROIs) in TMA core images from the discovery and validation cohort. An image pre-processing step is added after IHC staining deconvolution to avoid melanin pigmentation in single melanocytes or basal epidermis to be interpreted as Melan-A staining. During this step, upon the deconvolved DAB-CHANNEL image, a region growing morphological operation is applied to the mask to select clusters of melanoma cells stained with Melan-A. This approach is calibrated using melanoma tissue images from the discovery and validation cohorts with Melan-A and pigmentation. A detailed description of the steps involved for generating a Melan-A mask from an IHC stained TMA tissue spot is provided in Text S1. Figure S1 shows image examples of tissue cores stained with Melan-A from both cohorts as well as the respective masks. The Melan-A ROIs are further used as true binary labels for melanoma cells in the training and validation process.
- **Cell nuclei segmentation and feature extraction.** In the proposed method, advantage is taken from the H-CHANNEL deconvolution to highlight nuclei as bright spots from background. More importantly, to effectively segment clumped objects, CellProfiler contains a modular three-step strategy based on previously published algorithms [29], [30]. Once the nuclei were identified a mask of the corresponding nuclei was stored and this mask was used to measure their texture and morphological properties. A detailed list of these features is described in Table S1. This list includes features previously used for microscopy pattern recognition applications [31] as well as Haralick and Gabor texture features. We used the algorithms already implemented in CellProfiler [19] to compute these features, however, other commercial or publically available packages could be used.

### Definition of training and validation datasets

Three different datasets were generated to develop and validate the melanoma cell pattern recognition model.

1. Discovery dataset (**Melan-A_DISCOVERY**). Cell nuclei features extracted from images in the discovery dataset (n = 334) were used for feature selection and generation of a classification model. A total of 73 features (Table S1) were extracted from each nucleus in the training dataset (n = 369882). Given the unbalanced class (i.e. melanoma and non-melanoma) distribution of the training dataset and to avoid overfitting, nuclei were randomly selected from the full training dataset to derive a balanced class training dataset (n = 9353). A classification model was built using the balanced training dataset and internally validated on the remaining cell nuclei features of the full training dataset.
2. Hematoxylin validation dataset (**H_VALIDATION**). In order to test the reproducibility of the feature selection and classification model on consecutive TMA sections from the validation cohort, sections from melanoma tissue included in the discovery dataset were digitalized prior and post-immunostaining with Melan-A antibody. Stain deconvolution followed by nuclei segmentation and feature extraction was applied identically to the Melan-A stained sections.
3. Independent Melan-A validation dataset (**Melan-A_VALIDATION**). Cell nuclei features extracted from images in the validation dataset (n = 615) were used for independent validation of the classification model learned on the training dataset. Stain deconvolution followed by nuclei segmentation and feature extraction was applied to the validation Melan-A stained TMA sections. Melan-A stained sections in the discovery and validation cohort were stained and digitalized under same conditions. Using the Melan-A mask, a set of labeled nuclei (n = 386781) was identified (i.e. melanoma or non-melanoma) to test the melanoma classification model built on the discovery cohort.

### Feature selection

Given the unbalanced class (i.e. melanoma and non-melanoma) distribution of the Melan-A_VALIDATION, nuclei were randomly selected from the full training dataset to derive a balanced class training dataset (n = 6179). This feature set was merged with the random feature set from the Melan-A_DISCOVERY dataset. Nuclei features relevant for classification of melanoma and non-melanoma cell were evaluated on the merged class balanced dataset. Features were ranked based on the significance of the difference in expression levels (i.e. p-values) in the feature selection balanced dataset. After sorting them according to their rank, each feature was iteratively removed from Melan-A_DISCOVERY, balanced and the full Melan-A_VALIDATION dataset. After removal of a feature:

- The balanced Melan-A_DISCOVERY dataset was randomly split into training (66.5%) and internal validation (33.5%) datasets. A SVM model was learned on the training dataset and then validated in the testing part of the Melan-A_DISCOVERY dataset.
- A second independent validation was carried out on the balanced and the full Melan-A_VALIDATION datasets.
- The optimal feature set was the one yielding maximum predictive accuracy in the Melan-A_DISCOVERY and Melan-A_VALIDATION datasets.

Receiver operator curve analysis (ROC) was carried out to test the univariate predictive accuracy of each selected feature in the final optimal set. SPSS (version 18) was used to assess the significance of difference of mean expression of features values in the melanoma and non-melanoma cells. ROC analysis was also carried out using SPSS. Matlab and LIBSVM implementation of SVM were used to iteratively find the optimal feature set [32].

### Learning of a cell classification model

From all extracted morphological and texture features, the ones not selected in the previous step were filtered out from all training and validation datasets. The class balanced Melan-A_DISCOVERY dataset was used to develop a melanoma cell classification model. WEKA3 [33] a machine learning and data mining software (version 3.6.7) was used to derive a learning model and test the model on the validation datasets.

### Classification model construction and validation

Three classification methods were used: a probabilistic learner, Naïve Bayes (NB), a decision tree learner, random forest (RF) [34], [35] and SVM [36], [37]. These methods are applicable for different research problems, considering the specific advantages and disadvantages for each method. Naïve Bayes, random forest clustering and classification algorithms have the advantage of producing classification models that are easy to interpret. SVM have shown to be effective in deriving highly accurate classification models in digital pathology [20].

### Independent biomarker validation dataset

To validate the use of the melanoma cell classification model for IHC biomarker quantification a set of consecutive sections were cut from the validation cohort TMA and stained for the cell proliferation marker Ki67. TMA sections from the validation cohort were stained for Ki67 and digital images were captured as described above. From all tissue spot images, a subset (n = 270) were selected to be analyzed with the feature extraction pipeline described above and tested with the melanoma cell classification model. Using the melanoma classification model, all melanoma cells were identified and quantified as either positive or negative for KI67 staining. A trained user was also asked to blindly score the number of Ki67 positive and negative melanoma cells from each image in the Ki67 validation set. The results from the automated and manual quantification of Ki67 staining were cross-compared.

## Results

### Effect of variability in staining and image acquisition settings

**Figure 2:**
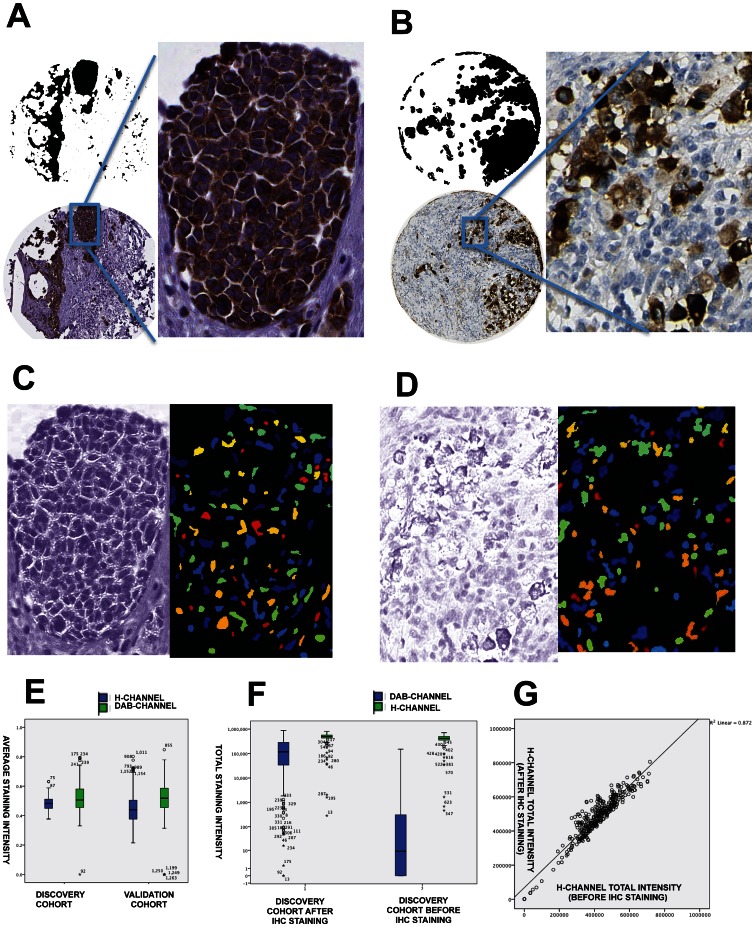
Melan-A and nuclei segmentation dependence on the staining variability in the three patient cohorts available. Using CellProfiler a Melan-A staining IHC positive mask is generated for TMAs sections from the discovery (**A**) and the validation (**B**) cohorts. In addition a second TISSUE mask was generated from the hematoxylin deconvolved channel (i.e. H-CHANNEL) highlighting all areas of the tissue were there was tissue present (**C–D**). Box and whiskers plot of average intensity of the DAB-CHANNEL and the H-CHANNEL in the discovery and the validation cohort shows similar values and no significant differences (**E**). On the segmented regions of interest (i.e. TISSUE and Melan-A) generated from the CellProfiler ruleset intensity of H-CHANNEL and DAB-CHANNEL was measured and distribution across the cohorts shows similar levels of total hematoxylin intensity (in the log_10_ scale), measured as sum of all intensity values within the segmented ROIs, but a significantly decrease of total intensity values of DAB in the Melan-A ROIs between the discovery and validation cohort with the hematoxylin counterstained cohort (**F**). Scatter plotting of total intensity values of the deconvolved H-CHANNEL for the same tissue samples images from the discovery cohort were same samples were once stained with counterstained with hematoxylin and then independently stained with Melan-A (**G**).

Optimally titrated primary antibodies for IHC and standardized hematoxylin counterstaining protocols were used throughout the study. However, due to the well-known variability of immunostaining assays, we tested how robust the stain deconvolution and cell nucleus segmentation was towards this variability. The color deconvolution algorithm was calibrated in the Melan-A_DISCOVERY cohort according to the previously published guidelines [28]. The resulting matrix of colorimetric calibration settings of Melan-A (Red  = 0.269, Green  = 0.570, Blue  = 0.776) and hematoxylin (Red  = 0.575, Green  = 0.681, Blue  = 0.453) was used throughout all the CellProfiler analysis rule sets. Using these pre-defined colorimetric settings, three melanoma TMA glass slides, digitalized independently, were analyzed with regard to the deconvolved H-CHANNEL and Melan-A-CHANNEL intensity variation (Figure 2A–B). After color deconvolution, each image channel was independently analyzed to identify all DAB positive cell nucleus (Figure 2C–D). There were similar levels of average DAB-CHANNEL ROIs intensity levels when comparing the discovery and validation cohorts (Figure 2E). In order to evaluate the accuracy of the color deconvolution method to estimate the optical density of DAB and hematoxylin, the glass slides from the discovery cohort were digitalized both prior to the immunostaining (hence with only the hematoxylin counterstain) and after immunostaining (Figure S2A). The CellProfiler ruleset was applied to both data sets and intensity levels were compared (Figure S2B). Paired-samples comparison showed an excellent correlation of segmented hematoxylin positive tissue area (Rho  = 0.832, p-value<0.001) as well as average hematoxylin intensity levels (Rho  = 0.837, p-value<0.001). Total intensity levels, measured as sum of intensity values of all pixels in the DAB and hematoxylin ROIs showed similar levels for hematoxylin. A significant difference of total DAB intensity levels was found (Figure 2F). The few outliers from the low levels of DAB observed from the discovery cohort prior to the immunostaining, after visual inspection were confirmed to be due to high levels of endogenous melanin pigment present in these samples. A linear regression analysis of the intensity levels further confirmed the near perfect correlation of the total intensity values of the H-CHANNEL in the discovery cohort prior and post immunostaining (Figure 2G). The CellProfiler rule set used to generate the DAB and hematoxylin ROIs, as well as extraction of the intensity values, can be found in the Text S2.

### Cell nucleus segmentation

Together with the calibration of the staining colorimetric properties, the calibration of the cell nucleus segmentation is a critical step prior to extraction of features describing the texture and morphological properties of the melanoma and non-melanoma cell nuclei. The challenge and objective of the cell nucleus segmentation, is to develop and calibrate tissue cell nucleus segmentation and feature extraction using the discovery cohort sample images and independently validate this segmentation approach unchanged in an independent cohort of samples. A detailed description of the CellProfiler rule set used to segment the nuclei is given in Text S1 and the code can be found in Text S3. Cell nuclei segmentation was validated for reproducibility by comparing the result of Melan-A_DISCOVERY with H_DISCOVERY cohorts and Melan-A_DISCOVERY with Melan-A_VALIDATION.

**Figure 3:**
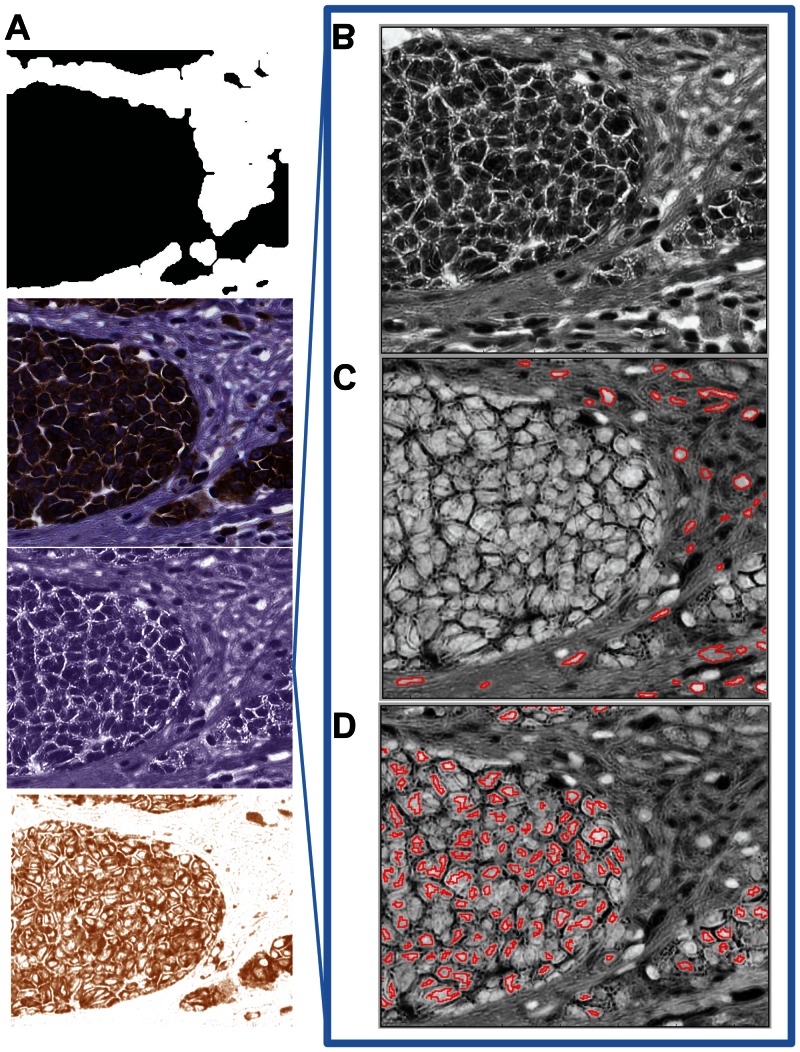
Cell nuclei segmentation of Melan-A stained TMAs tissue spot images. The CellProfiler ruleset take as input the original IHC stained tissue core image as well as the Melan-A binary mask (**A**). (**B**) In the next step the corresponding H and DAB channel images are deconvolved and only the H-CHANNEL image is further processed for cell nucleus segmentation. Initially H-CHANNEL image is pre-processed to sharpen the image and highlight object of interest (i.e. cell nucleus). Using the binary Melan-A mask in **A,** non-melanoma (**C**) and other types of cells (**D**) nucleus are segmented out (highlighted in red in panel **C** and **D**) using the robust background segmentation method in order to extract morphological and texture features.

**Figure 4:**
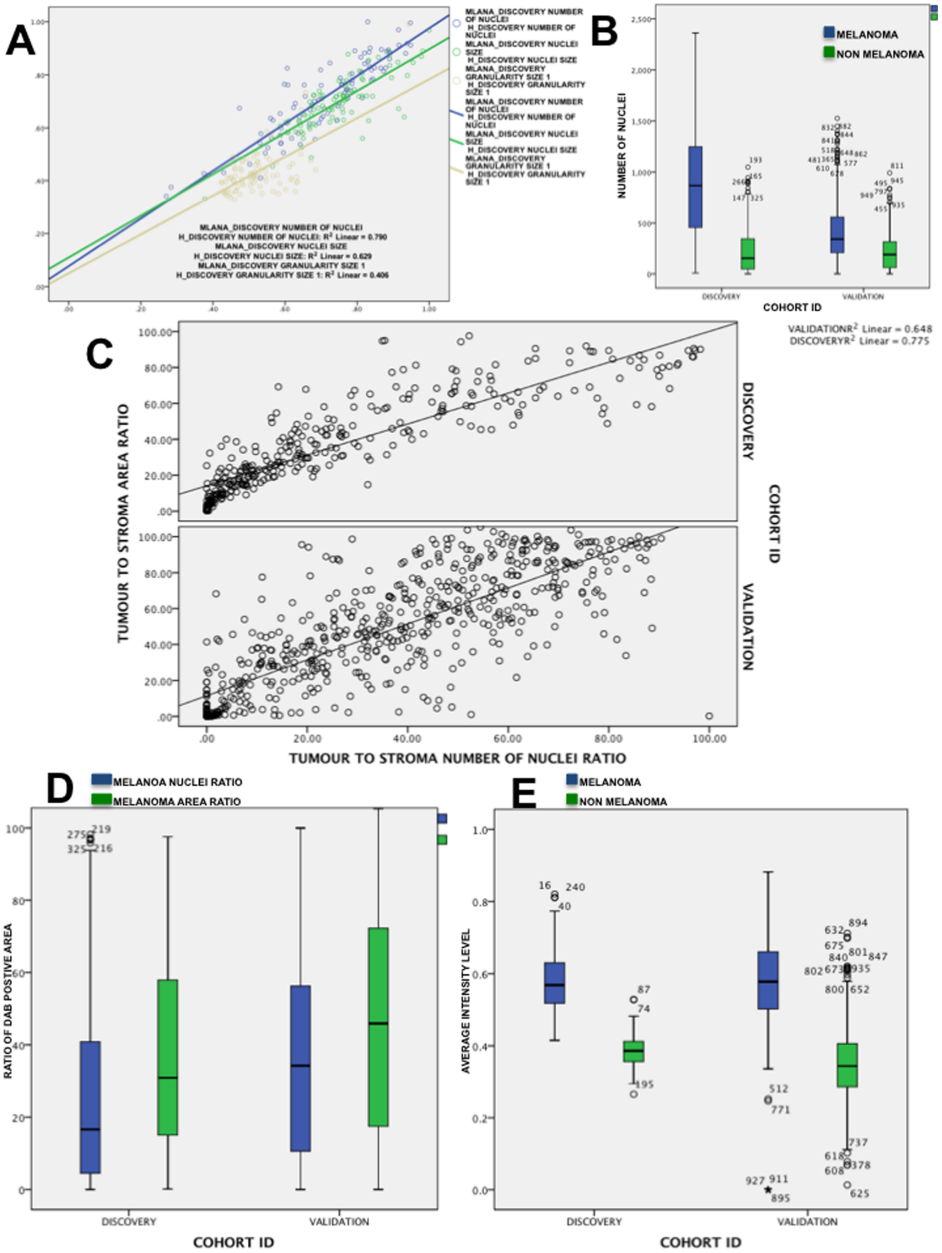
Cell nuclei segmentation on the discovery and the validation cohort. Using the Melan-A binary masks, melanoma and the remaining cell nucleus are selectively segmented from all Melan-A stained tissue spot images in the discovery and the validation cohorts. (**A**) Linear regression analysis of average nuclear size, total number of nuclei and average granularity of size 1 features on the same tissue samples on the Melan-A_DISCOVERY and H_DISCOVERY cohorts after standartisation (divided by the maximum value and scaled at minimum value of 0 and maximum value of 1). (**B**) Average number of segmented melanoma (green bars) and other type of cell nuclei (blue bars) per tissue spot image in the MELAN-A_DISCOVERY and Melan-A_VALIDATION cohort. (**C**) Comparing the ratio of Melan-A positive tissue area ratio to the ratio of Melan-A positive cell nuclei ratio detected by the approach, the scatter plot shows how correlated the ratios are in both cohorts. (**D**) Ratio of Melan-A stained tumor area towards total amount of present tissue (green bars) and the ratio of melanoma cell nuclei (as defined by the Melan-A mask) to total number of detected nuclei (blue bars) in both cohorts. (**E**) Average intensity levels after stain deconvolution of IHC Melan-A intensity in Melan-A mask highlighted areas (blue bars) compared to the average hematoxylin intensity (green bars) in both cohorts.

CellProfiler was used to segment nuclei in images from the H_DISCOVERY and Melan-A_DISCOVERY cohorts (Figure S3A–B). After H-CHANNEL was deconvolved in the original images (Figure 3A–B), the nuclei were identified using the steps described in Text S1 in the melanoma tumor stroma (Figure 3C) and tumor nest mask (Figure 3D). Nuclei morphological and texture features were measured using the nuclei mask in H-CHANNEL (Figure S2B). Comparison of features extracted in images of same tissue samples (Figure S2A) showed an excellent correlation of number of nuclei identified in each of the paired samples (Rho  = 0.833, p-value<0.001) as well as average nuclear size (Rho  = 0.750, p-value<0.001) (Figure 4A). One of the top texture features characterizing tumor cell nuclei and recently reported to distinguish them from the normal tumor stroma cells nuclei is granularity [10], [38]. We compared granularity as feature extracted using CellProfiler (structuring element of radius 1 pixel) between H_DISCOVERY and Melan-A_DISCOVERY cohorts. Again, the correlation of the granularity feature extracted from paired samples showed a good correlation (Rho  = 0.429, p-value<0.001) (Figure 4A).

In a second step we validated the cell nuclei segmentation by comparing results of running the same cell nuclei segmentation ruleset, between the Melan-A_DISCOVERY and Melan-A_VALIDATION cohorts. Accordingly to proportional differences in size of tissue spots (i.e. 1 mm and 0.6 mm), the numbers of detected melanoma and non-melanoma cell nuclei were similar between the Melan-A_DISCOVERY and Melan-A_VALIDATION cohorts (Figure 4B). This is also confirmed by the linear regression analysis of the ratio of DAB positive to total amount of tissue present with ratio of melanoma cells to non-melanoma cell nuclei in the Melan-A_DISCOVERY (R^2^ = 0.775) and Melan-A_VALIDATION (R^2^ = 0.648) (Figure 4C). When looking at the distribution of melanoma and non-melanoma ratio of nuclei or the total area of Melan-A positive tissue area, similar levels were observed in both cohorts (Figure 4D). Finally, analysis of DAB staining intensity in the DAB-CHANNEL of Melan-A IHC positive ROIs and H-CHANNEL intensity levels shows very different and significant different intensity levels for both cohorts further confirming the accuracy of detecting melanoma and non-melanoma cell nuclei in both cohorts (Figure 4E).

### Feature selection

Features were initially ranked from the initial full set of 73 morphological and texture features (Table S1), followed by the identification of the optimal subset of features for distinguishing melanoma from non-melanoma nuclei. In order to avoid overfitting, we proceeded by ranking the features on a class-balanced (i.e. melanoma and non-melanoma cells) random subset of the Melan-A_DISCOVERY and Melan-A_VALIDATION data sets (n = 15532) (Table 1). Difference of mean level of each feature for melanoma and non-melanoma cell nuclei was tested (t-test on the equality of the mean) on a randomly drawn sub-sample (5%, n = 769) of cell nuclei to avoid high significance associated with small feature changes due to sample size. All features were further ranked according to the p-value of equality of the mean levels between melanoma and non-melanoma cell nuclei (Table S1). A scatter plot of all the nuclei from the feature selection dataset (n = 15532) following the top five most differently expressed features (i.e. lowest p-value) showed good discrimination of melanoma and non-melanoma cells following these features (Figure S4A).

**Table 1:** Distribution of number images and identified cell nuclei in the training and validation data sets.

Dataset	# tumour cells	# other cells	# total cells	# images
training full	76696	293186	369882	334
training balanced	4662	4691	9353	334
validation full	128929	257852	386781	615
validation balanced	3077	3102	6179	615
validation Hematoxylin	unknown	unknown	43799	90

**Figure 5:**
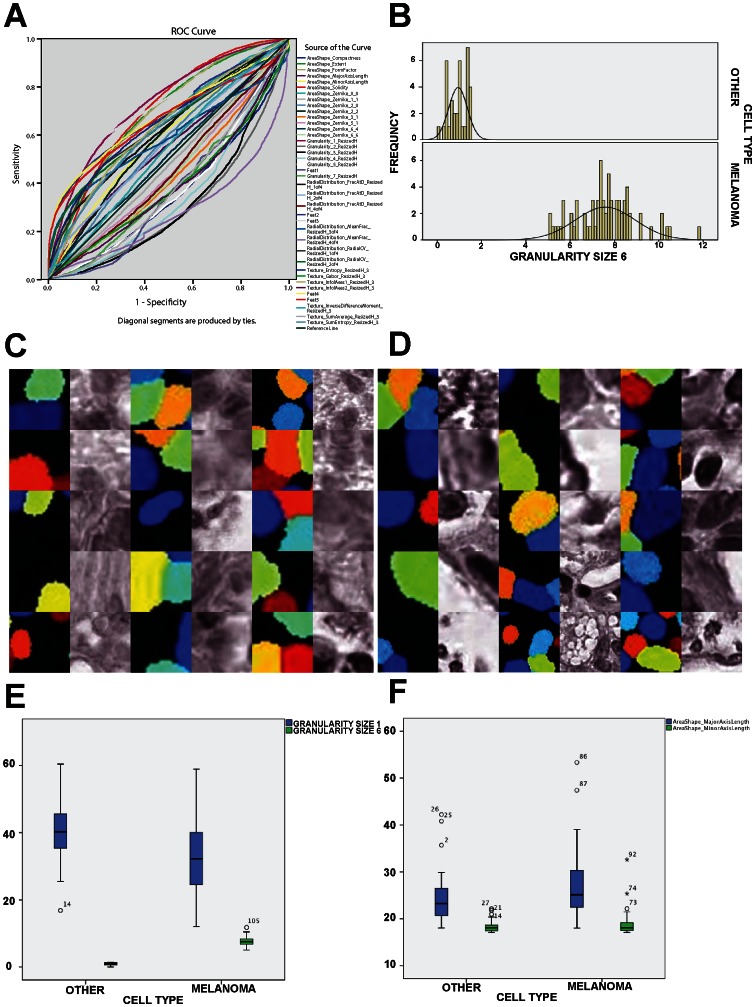
Cell nuclei feature discrimination. From the balanced pooled feature set from the Melan-A_DISCOVERY and Melan-A_VALIDATION dataset, selected features from the univariate analysis were tested using Receiver-Operator-Curve (ROC) analysis to show the performance of a binary classifier (i.e. melanoma or non-melanoma) system as its discrimination threshold is varied for each of the selected features (**A**). To illustrate the differences in granularity (top discriminative feature from the ROC analysis), a representative set of melanoma and non-melanoma nuclei were selected from the discovery TMA and the histogram of the granularity feature (i.e. six pixel radius) values from each set is shown in **B.C–D**) Top texture feature (i.e. granularity feature with structure element of six pixel radius) in melanoma cells (**C**) and non-melanoma cells (**D**) with cell nucleus masks shown using different colored segmentation labels to differentiate nucleus of touching cells. **E**) Box-plot showing distribution of top granularity features with variable structure element size (i.e. six and two pixel radius) that show significant difference (p-value <0.001) in expression values between melanoma and non-melanoma cells. **F**) Comparison of major and minor axis of bounding ellipse around the nuclei of melanoma and non-melanoma cells shows no significant difference, showing no correlation of cell nucleus segmentation size and granularity feature as well as no cell selection bias for the evaluation of the granularity feature.

Iteratively from the full feature set, starting from the less differently expressed features (i.e. high p-values), a reduced feature training (66.5% of class-balanced Melan-A_DISCOVERY), testing (33.5% class-balanced Melan-A_DISCOVERY) and independent validation (class-balanced and full Melan-A_VALIDATION) datasets were generated. A LibSVM melanoma cell classification model was build in the training dataset and tested in both independent validation datasets (Figure S4B). The cubic interpolation line fitted on the classification accuracy values of the full independent Melan-A_VALIDATION datasets (blue dots) (Figure S4B) was used to find the set of features for which the maximum accuracy is reached and equation of the polynomial fit generated (i.e. 34 features, 86.4%). From the balanced merged feature set (i.e. Melan-A_DISCOVERY, Melan-A_VALIDATION class balanced datasets), selected features from the optimal feature set were tested using ROC analysis and area under the curve was measured for each feature as performance indicator (Table S2). ROC analysis shows the performance of a binary classifier (i.e. melanoma or non-melanoma) system as its discrimination threshold is varied for each of the selected features (Figure 5A). ROC analysis of top most discriminant features also shows how relevant these features are to discriminate between melanoma and non-melanoma cells with area under the curve (AUC) values ranging from 0.663 to 0.734 (Figure S4C), also confirming the granularity with a structuring element of radius one pixel being the top most discriminative feature (AUC = 0.734).

To illustrate the differences in granularity a representative set of melanoma (n = 30) and non-melanoma nuclei (n = 30), showing highest differences of granularity were selected from the balanced Melan-A_DISCOVERY cohort (Figure 5B). Images of the deconvolved H-CHANNEL of the melanoma (Figure 5C) and non-melanoma (Figure 5D), for these cell nuclei using the Melan-A binary masks also show distinctive granularity features. Difference of mean values for the top two granularity feature (size of structuring element radius 1 and 6 pixels) between the melanoma and non-melanoma cell set showed also to be significant (p-value<0.001) (Figure 5E) as well as negatively correlated (Rho  = −0.337). Comparison of size (number of pixels) for the major and minor axis of bounding ellipse around the nuclei (Figure 5F) of melanoma and non-melanoma cells showed no significant difference, as well as no significant correlation with the granularity feature values.

### Classifier training

**Figure 6:**
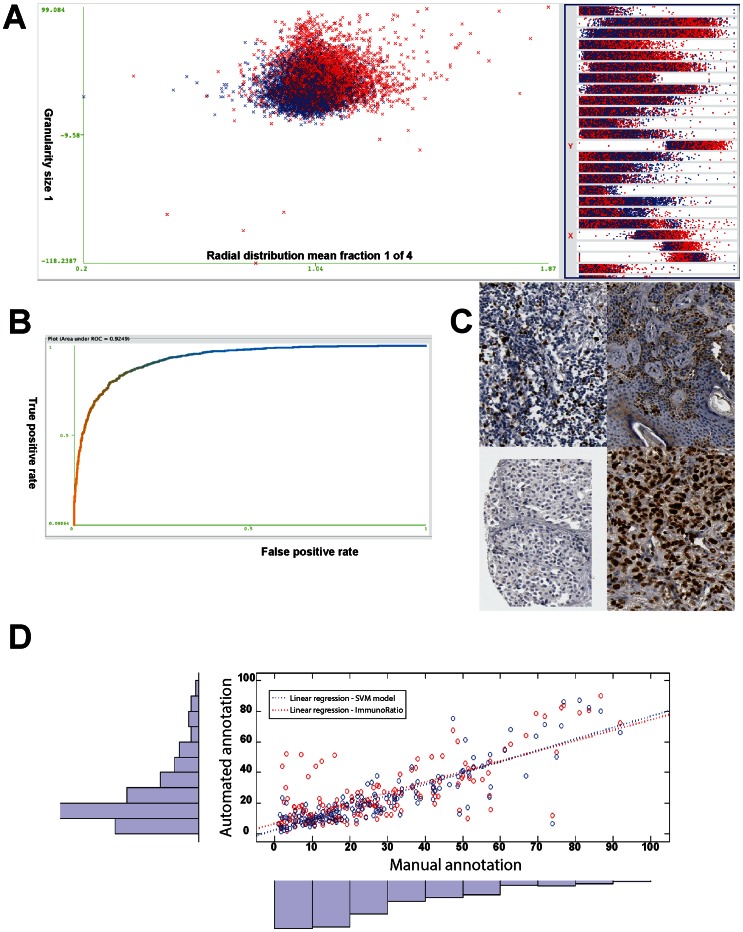
Classifier training and validation. An overview of the cell nuclei in the balanced Melan-A_DISCOVERY dataset following the optimal feature set (i.e. 34 features selected in the previous step) (**A**). A SVM classifier is build using data from the training set (66.5%) and tested on the remaining cases (33.5%). To test the robustness of the classifier, during testing Weka varies the threshold (by default set to 0.5 and the label for the nuclei would be the class with probability higher then 50%) on the class probability estimates from LibSVM for melanoma and non-melanoma resulting in different classification labels for the cell nuclei in the test set (**B**). The SVM classifier derived from the balanced Melan-A_DISCOVERY is then validated in 270 randomly selected tissue samples from the validation cohort stained with Ki67 antibody (**C**). Linear regression analysis is used on the automated counting of ratio of melanoma cells expressing Ki67 by the SVM-based model (blue dotted line) and ImmuRatio (red dotted line) and the same ratio exactly evaluated by an experienced human annotator (**D**).

To select the best fitted classifier for the classification of melanoma and non-melanoma cell nuclei a Naïve Bayes Network classifier (NBN), a Random Forest classifier (RF) and a Support Vector Machine (SVM) classifier were trained on the two-thirds (66.5%) of the balanced Melan-A_DISCOVERY and then evaluated internally on the remaining of the balanced Melan-A_DISCOVERY data set (33.5%)(Figure 6A). Validation of the classifiers models show similar performances for SVM and RF and a clear improvement compared to NBN (Table 2). However, the independent validation of the SVM and RF supervised models in full Melan-A_VALIDATION dataset shows that SVM outperforms RF (i.e. average out performance 1.7%) indicating that SVM is more appropriate for learning a better generalizable melanoma classification model.

**Table 2:** Performance evaluation on divers supervised classifiers upon the balanced Melan-A_DISCOVERY and Melan-A_VALIDATION cohorts.

Data set	Classifier	Support Vector Machine (SVM)	Random Forest Clustering (RFC)	Bayes Network (BN)
**BALANCED Melan-A_DISCOVERY**	TP Rate	0.85	0.867	0.79
	FP Rate	0.15	0.133	0.21
	Precision	0.85	0.867	0.793
	Recall	0.85	0.867	0.79
	F-Measure	0.85	0.867	0.79
	ROC Area	0.925	0.942	0.861
**BALANCED Melan-A_VALIDATION**	TP Rate	0.914	0.877	0.834
	FP Rate	0.105	0.123	0.166
	Precision	0.891	0.887	0.842
	Recall	0.914	0.877	0.839
	F-Measure	0.913	0.883	0.929
	ROC Area	0.964	0.952	0.834

### Classifier validation

LibSVM [32] was selected to generate a probabilistic logistic classification model to control the label probability threshold in order for true positive (TP) rate of melanoma nuclei (i.e. by default set to 50%). A LibSVM model was generated on the training part of the balanced Melan-A_DISCOVERY dataset (66.5%) and tested on the remaining set of nuclei (33.5%). WEKA was used to illustrate the classifier prediction trade-offs that can be obtained by varying the threshold value between classes (i.e. melanoma and non-melanoma). The resulting classification performance on the test set was used for ROC curve analysis to show the robustness of the classifier with regard to the class probability boundary (AUC = 0.924)(Figure 6B). The classifier developed in the Melan-A_DISCOVERY dataset was tested independently in the Melan-A_VALIDATION data set (Table 2). Additionally, the same classifier was tested on samples stained only with hematoxylin from the discovery cohort (i.e. H_DISCOVERY). The results were visually inspected by a trained pathologist (FP) and deemed to be accurate with regard to melanoma cell classification. Example of input from the H_DISCOVERY and the corresponding output from the SVM melanoma classification model are shown in Figure S5A–D.

### Classifier validation for biomarker quantification

One of the goals, for developing image pattern recognition models as described, is to use these models for recognizing melanoma cells in images of tissue sections from melanoma specimens. In this particular study the goal was to identify melanoma cells with high accuracy and reproducibility in both the dicovery and validation cohort. Once the recognition of the melanoma cells in these sections is possible, then the deconvolved DAB-CHANNEL could be used to quantitate the expression levels of any given protein candidate biomarker of interest (Figure S6A). The developed SVM-based melanoma classification model was further validated for general biomarker discovery in a consecutive section of the validation cohort stained with Ki67. Ki67 is a well-known biomarker for cell proliferation, earlier described in the discovery cohort [39]. Ki67 expression in tumor cells has been suggested as a prognostic indicator for cancer patients, including melanoma [40] (Figure 6C). A set of randomly selected tissue spot images (n = 270) in the discovery cohort was assesed by manual evaluation and scoring (JB). The amount of melanoma cells were manually counted and the ratio of these cells expressing the Ki67 protein was calculated. The same images were subjected to an adapted CellProfiler rule set (Text S3), extended with additional analysis steps to quantitate the deconvolved DAB-CHANNEL for Ki67 expression in a cell-by-cell basis (Figure S6B). The SVM pattern recognition model was applied to the texture and morphological features extracted from selected tissue spot images (n = 270) and the total amount of melanoma cells as well ratio of these cells expressing Ki67 was quantified (Figure S6C). A linear regression analysis of the manual and automated quantification results of Ki67 showed a high level of agreement between the scores obtained by manual counting and the automated SVM-based melanoma classification and biomarker quantification approach (R^2^ = 0.745, Rho  = 0.831) (Figure 6D). Automated results were also compared to ImmunoRatio, a previously published method for quantification of Ki67 in IHC stained TMAs tissue spots [41] (Figure 6D). Comparison of Immunoratio Ki67 ratio to the SVM-based approach by us developed showed excellent agreement (Rho  = 0.776) and also good agreement with the manual annotation (Rho  = 0.662).

## Discussion

The aim and purpose of the present work was to develop an algorithm for automated quantification of IHC-determined protein expression in melanoma tumor specimens and to attain a strategy that could be further extended to other solid tumor types. All the tools used to generate the obtained data and utilize the pattern recognition model are publically available, making this system valuable for use in the research community. Previously described automated methods such as AQUA, take advantage of known biological markers, e.g. cytokeratin markers and have been extensively used in fluorescence-based pathology assays [13], [42]. However, up-to-date no functional method has been proposed for use with optical bright-field high throughput microscopy pathology applications. The presented approach differs from other commercially available packages and previously proposed supervised methods, since a molecular marker is used to objectively select data for training purposes. Additionally, previously described methods for use with digital pathology pattern recognition applications, have focused on laborious image object identification by skilled pathologists, followed by the measurement of texture and morphological features of single or group of pixels with similar texture or color properties [9], [10], [43]. Methods characterizing epithelial nuclear characteristics, such as size, color and spatial distribution in order to distinguish tumor and lymphocyte infiltration has shown to be effective in solid tumors such as breast cancer [11], [44]. Here, we propose an extension of this work that learns to identify texture patterns of true biological objects such a cell nuclei instead of pixels. A high quality counterstain is helpful, however, we show that it does not have to be specifically controlled for automated analysis.

To our knowledge this is the first time such a cell-type specific marker has been proposed to develop a supervised machine learning classification model for digital pathology. In contrast, to previous methods the learning and validation process of the method proposed here is fully automated with regard to the training and validation. Once the specificity of the antibody for Melan-A and digitalization is approved there are no requirement for image filtering to ensure high-quality TMA images for the training. One of the challenges in developing automated methods for pattern recognition in digital pathology is the selection of robust methods of segmenting objects that are biologically relevant (e.g. nuclei, cells or vessels) and secondly selecting features describing biologically relevant changes of morphology or texture at the micro or macro tissue level [45]. Both segmentation and feature selection are affected by non-biologically relevant variability in staining and image acquisition settings [17]. Independent validation of the presented SVM-based melanoma model on digital slides stained and digitalized at different times shows robustness with regard to such variations (Table 2).

In contrast to previous approaches, our system proposes the use of a limited number of texture and morphology features (n = 73) that could be measured in a reasonable amount of time (average of 2 minutes for 1 mm diameter tissue core image) allowing such a method to be of practical consideration for high throughput assays. Further reduction of these features shows to improve both internal classification during the training and validation set as well as independent validation on the a second independent cohort of patients (Figure S4B). A key computational aspect of the approach proposed here is use of unbiased data-driven approach to identify morphologic and texture features relevant for discrimination of melanoma and non-melanoma cell nuclei. We have earlier shown that this discovery-based approach is applicable in the study of cancer morphology and texture changes from microscopic images of patient samples [43]. Selection of cell nuclei elongation in the top most discriminative features for melanoma and non-melanoma in the present study as well as selection of this feature for discrimination of infiltrating lymphocyte and tumor cell nuclei in breast cancer [44] indicates the possibility of such method to be scalable to the analysis of other solid tumor types.

Our work demonstrates the ability to apply publically available image analysis tools together with a machine-learning framework to build a powerful pathology image–based pattern recognition model from very small samples of a solid tumor. Use of cell nuclei as seeds for quantifying whole cell biomarker expression levels in melanoma cells is demonstrated by validating the SVM-based model in consecutive sections from the validation cohort. The in-build features of the cell nucleus and cell outer border segmentation also allow for discrimination of biomarkers expression with regard to these subcellular compartments (Figure S6A). All analysis pipelines used to generate the data presented here are available in the attached document. Since antibody-based proteomics occupies a key role in the cancer biomarker discovery and validation pipeline, such versatile automated tools would facilitate the high-throughput translation of candidate biomarkers in melanoma from the research bench to clinical implementation [1]. Although all images used in our study came from melanoma TMAs digitalized glass slides, inclusion in the training and validation of large collection of TMAs melanoma patient samples (n = 949) allows the derived classification model to capture general changes of melanoma and non-melanoma cells in TMAs.

As a conclusion, here we propose a method for general use in biomarker melanoma cell pattern recognition and melanoma cell specific biomarker quantification. Use of TMAs for the training and validation is both a strength and a limitation of this study. Since we use a large number of samples to train and validate the melanoma cell classification model we propose this method for general use with TMAs. In order to extend the model for use with full glass slides, a further stratification of the non-melanoma cells and extension of the feature set to allow to capture differences with the new cells types are needed. As cell- and tumor-type specific protein expression is exceedingly rare [46], developing new antibodies with high specificity will remain a challenge. However, our strategy and results from the image processing and machine learning pipeline used with melanoma TMA images, suggest that this approach could be adapted and retrained with digital pathology data from other solid tumor types.

## Supporting Information

Figure S1 **Melan-A mask generation.** Using CellProfiler a Melan-A positive mask is generated for TMAs sections from the discovery (**A**) and the validation (**B**) cohorts. Melan-A binary masks (**C, D**) generated from the CellProfiler ruleset highlight in black, areas where there is Melan-A staining and with with white, IHC staining negative and slide background.
(TIF)

Figure S2 **Stain color deconvolution.** Figure shows deconvolution of DAB from Hematoxylin channels in examples of tissue spot images from the discovery and validation cohort stained only with hematoxylin and counterstained with hematoxylin and stained with an antibody against Melan-A (**A**). Bottom panel (**B**), shows the respective images of the hematoxylin channel after using the color decovolution algorithm (only the hematoxylin deconvolved channel shown).
(TIF)

Figure S3 **Validation of cell nuclei segmentation in the H_DISCOVERY and Melan-A_DISCOVERY cohorts.** (**A**) Images of the same tissue samples from the H_DISCOVERY and Melan-A_DISCOVERY cohorts are analysed using the cell nuclei segmentation CellProfiler ruleset (Data file S2). From each image all nuclei present are segmented and masks used to measure texture and morphological properties of the H-CHANNEL of each nuclei (**B**). H-CHANNEL after deconvolution of original images (**A**) is mapped using a false colors heat color map, encoding H-CHANNEL values from low (in blue) to high (yellow-to-red) (**C**).
(TIF)

Figure S4 **Optimum feature set selection. Iteratively from the full feature set, starting from the less differently expressed features, reduced feature training, testing and independent validation datasets (green dots) were generated.** A SVM melanoma cell classification model was trained (66.5% of data) and tested (33.5% of data) as well as independently validated in both the balanced and full Melan-A_VALIDATION datasets (**A**). A cubic interpolation line was fitted on the classification accuracy values of the full independent Melan-A_VALIDATION datasets (blue dots) and equation of the polynomial fit generated to find the number of feature for which the maximum accuracy is reached (i.e. 34 features, 86.4%) (**A**). Scatter plot of melanoma and non-melanoma cells from the balanced Melan-A_DISCOVERY and Melan-A_VALIDATION datasets following the top five most significantly expressed features (**B**). ROC analysis shows also how relevant, the top five most differently expressed feature, are to discriminate between melanoma and non-melanoma cells with AUC values ranging from 0.663 to 0.734 (**C**).
(TIF)

Figure S5 **Validation of the melanoma classification model.** The SVM model learned in the Melan-A_DISCOVERY is validated in the H_DISCOVERY cohort with the same samples only counterstained with hematoxylin (**A**). Images of paired tissue samples from Melan-A_DISCOVERY cohort (**B**) and the Melan-A_MASK (**C**) extracted by the CellProfiler ruleset, were compared to the automated melanoma (marked in Red) and non-melanoma cell nuclei (marked in Blue) in the images from H_DISCOVERY cohort (**A**).
(TIF)

Figure S6 **Validation of the melanoma classification model for general biomarker quantification.** Using the CellProfiler ruleset described in Data file S3, the nucleus of the cell (black cavity) is extracted from the original immunostained image and the cell is classifier as a melanoma or non-melanoma cell (**A**). Furthermore based on the deconvolved DAB-CHANNEL the boundary of the cell cytoplasm is fixed on the gradient of the staining pattern outside the nucleus and the cell defined as immunostained positive (annotated in red) or negative (annotated in blue) (**A**). This ruleset is further applied to two tissue core images from the validation melanoma TMA immunostained against Ki67 with a ratio of melanoma cells positively stained greaten then twenty per cent (**B**) and less then twenty per cent (**C**).
(TIF)

Table S1 **Descriptive statistics of all texture and morphological measurements made by CellProfiler modules extracted from non-melanoma and melanoma cell nuclei in the discovery TMA.** Last 3 columns summarise the univariate T-test of equality of means with a * denoting all significant features (p-value<0.05) and ** denoting the top most discriminative features (highlighted in bold).
(XLSX)

Table S2 **Receiver operator curve analysis of the features in the optimal feature set.**
(XLSX)

Text S1 **Schematic overview of the different steps of the image analysis pipeline starting from color deconvolution to the quantification of the immunostaining intensity and ratio of positive tumor cells.**
(DOC)

Text S2 **Source code of the CellProfiler ruleset used to segment the Melan-A and hematoxylin regions of interest from the original IHC images.**
(TXT)

Text S3 **Source code of the CellProfiler ruleset used to segment the melanoma and non-melanoma cell nuclei from the original IHC images.**
(TXT)
