# Supplementary figures and images for: A Texture Based Pattern Recognition Approach to Distinguish Melanoma from Non-Melanoma Cells in Histopathological Tissue Microarray Sections
Source: PLoS One. 2013 May 17;8(5):e62070. doi: 10.1371/journal.pone.0062070 (PMC3656869; doi:10.1371/journal.pone.0062070)

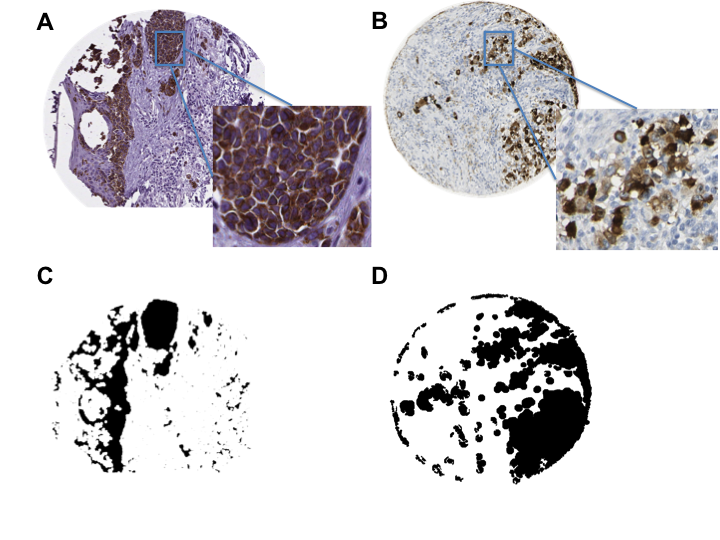

Supplement: Figure S1 — Melan-A mask generation. Using CellProfiler a Melan-A positive mask is generated for TMAs sections from the discovery (A) and the validation (B) cohorts. Melan-A binary masks (C, D) generated from the CellProfiler ruleset highlight in black, areas where there is Melan-A staining and with with white, IHC staining negative and slide background. (TIF) [file pone.0062070.s001.tif]

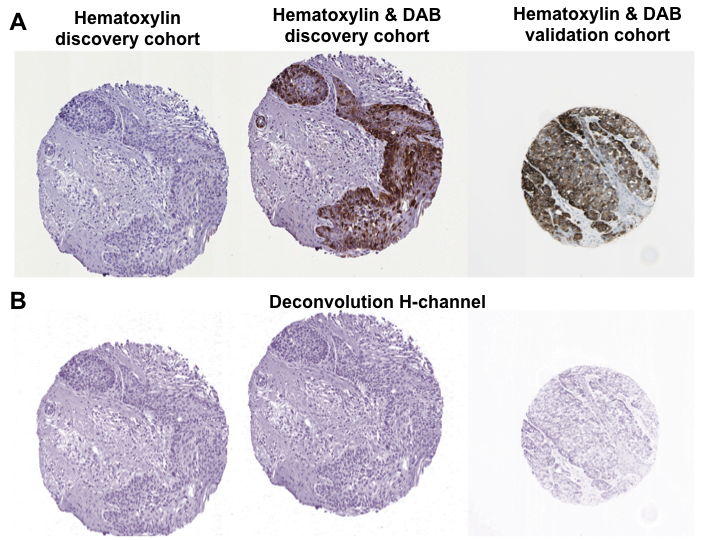

Supplement: Figure S2 — Stain color deconvolution. Figure shows deconvolution of DAB from Hematoxylin channels in examples of tissue spot images from the discovery and validation cohort stained only with hematoxylin and counterstained with hematoxylin and stained with an antibody against Melan-A (A). Bottom panel (B), shows the respective images of the hematoxylin channel after using the color decovolution algorithm (only the hematoxylin deconvolved channel shown). (TIF) [file pone.0062070.s002.tif]

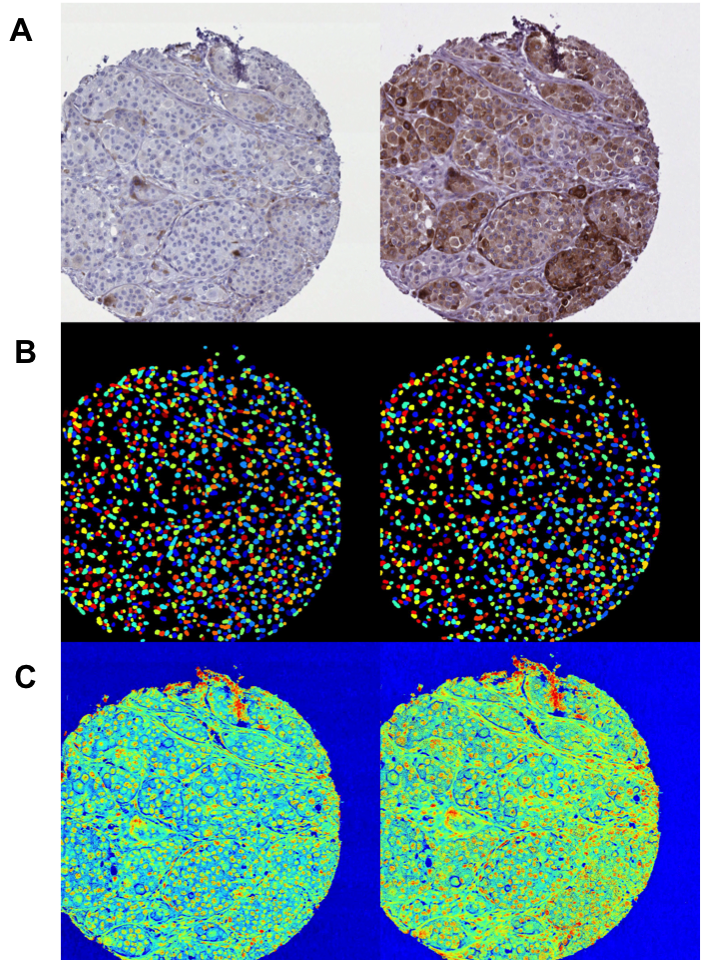

Supplement: Figure S3 — Validation of cell nuclei segmentation in the H_DISCOVERY and Melan-A_DISCOVERY cohorts. (A) Images of the same tissue samples from the H_DISCOVERY and Melan-A_DISCOVERY cohorts are analysed using the cell nuclei segmentation CellProfiler ruleset (Data file S2). From each image all nuclei present are segmented and masks used to measure texture and morphological properties of the H-CHANNEL of each nuclei (B). H-CHANNEL after deconvolution of original images (A) is mapped using a false colors heat color map, encoding H-CHANNEL values from low (in blue) to high (yellow-to-red) (C). (TIF) [file pone.0062070.s003.tif]

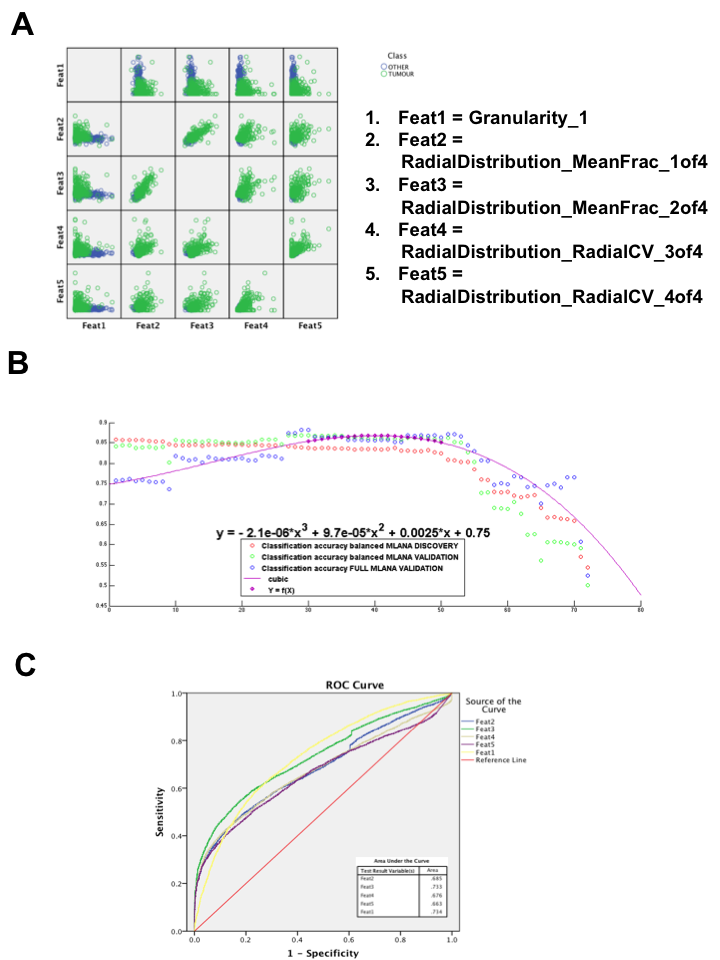

Supplement: Figure S4 — Optimum feature set selection. Iteratively from the full feature set, starting from the less differently expressed features, reduced feature training, testing and independent validation datasets (green dots) were generated. A SVM melanoma cell classification model was trained (66.5% of data) and tested (33.5% of data) as well as independently validated in both the balanced and full Melan-A_VALIDATION datasets (A). A cubic interpolation line was fitted on the classification accuracy values of the full independent Melan-A_VALIDATION datasets (blue dots) and equation of the polynomial fit generated to find the number of feature for which the maximum accuracy is reached (i.e. 34 features, 86.4%) (A). Scatter plot of melanoma and non-melanoma cells from the balanced Melan-A_DISCOVERY and Melan-A_VALIDATION datasets following the top five most significantly expressed features (B). ROC analysis shows also how relevant, the top five most differently expressed feature, are to discriminate between melanoma and non-melanoma cells with AUC values ranging from 0.663 to 0.734 (C). (TIF) [file pone.0062070.s004.tif]

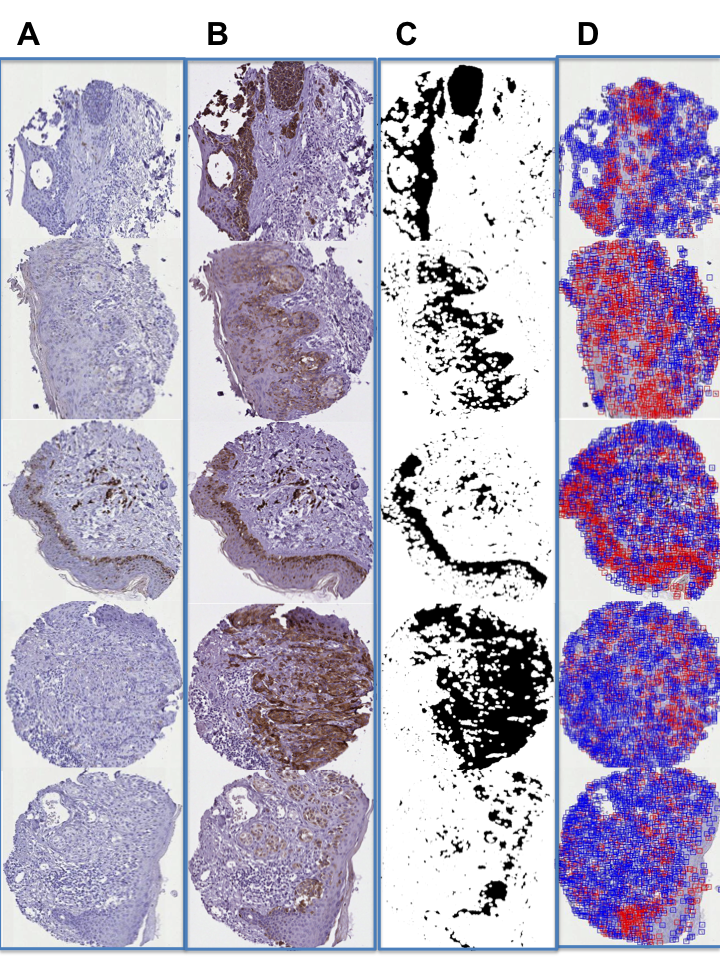

Supplement: Figure S5 — Validation of the melanoma classification model. The SVM model learned in the Melan-A_DISCOVERY is validated in the H_DISCOVERY cohort with the same samples only counterstained with hematoxylin (A). Images of paired tissue samples from Melan-A_DISCOVERY cohort (B) and the Melan-A_MASK (C) extracted by the CellProfiler ruleset, were compared to the automated melanoma (marked in Red) and non-melanoma cell nuclei (marked in Blue) in the images from H_DISCOVERY cohort (A). (TIF) [file pone.0062070.s005.tif]

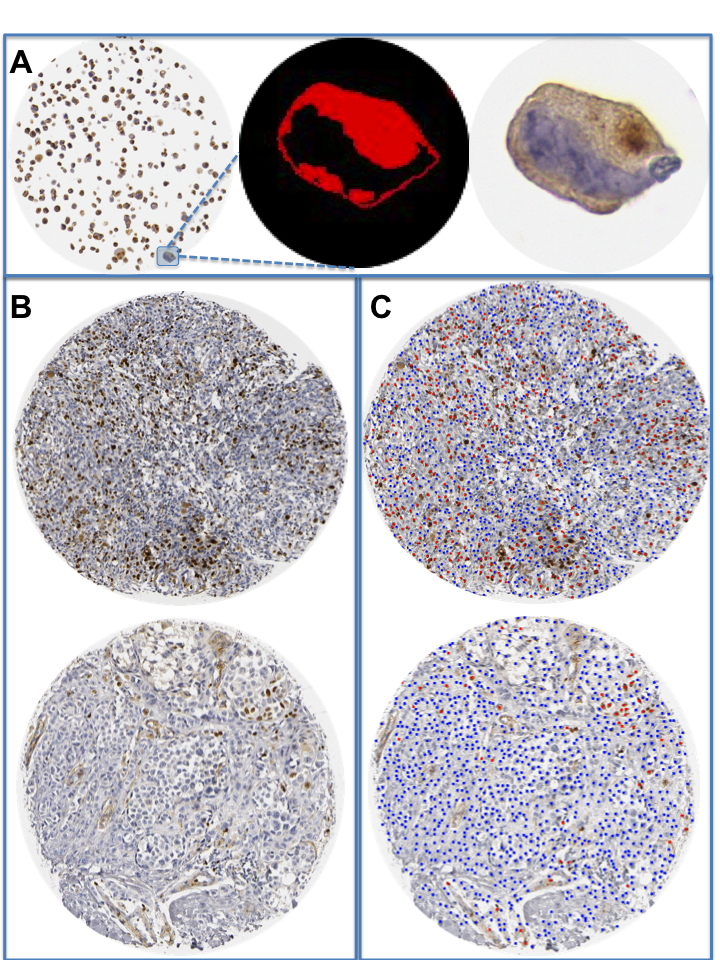

Supplement: Figure S6 — Validation of the melanoma classification model for general biomarker quantification. Using the CellProfiler ruleset described in Data file S3, the nucleus of the cell (black cavity) is extracted from the original immunostained image and the cell is classifier as a melanoma or non-melanoma cell (A). Furthermore based on the deconvolved DAB-CHANNEL the boundary of the cell cytoplasm is fixed on the gradient of the staining pattern outside the nucleus and the cell defined as immunostained positive (annotated in red) or negative (annotated in blue) (A). This ruleset is further applied to two tissue core images from the validation melanoma TMA immunostained against Ki67 with a ratio of melanoma cells positively stained greaten then twenty per cent (B) and less then twenty per cent (C). (TIF) [file pone.0062070.s006.tif]
