# Supplementary material for: A Texture Based Pattern Recognition Approach to Distinguish Melanoma from Non-Melanoma Cells in Histopathological Tissue Microarray Sections
Source: PLoS One. 2013 May 17;8(5):e62070. doi: 10.1371/journal.pone.0062070 (PMC3656869; doi:10.1371/journal.pone.0062070)
Supplement: Text S1 — Schematic overview of the different steps of the image analysis pipeline starting from color deconvolution to the quantification of the immunostaining intensity and ratio of positive tumor cells. (DOC) [file pone.0062070.s009.doc]

1. **Melan-A mask generation.** This ruleset aim at generating a Melan-A binary mask from the image of a TMA tissue spot stained with a Melan-A antibody.
   1. **UnmixColors. Although, default values are provided for various stains, it is advised to calibrate this vectors because of the know variability colorimetric properties of various chromagens, in particular diaminobenzidine (DAB) and hematoxylin (H) . From the discovery cohort tissue core images of the same patient stained only with H and H&DAB were used to calibrate RGB colorimetric properties of each stain (Figure S2A-B). To perform separation of the DAB from H, we used the following vectors as input for CellProfiler:**

|  | **H** | **DAB** |
| --- | --- | --- |
| **Red** | **0.5749** | **0.2681** |
| **Green** | **0.6810** | **0.5703** |
| **Blue** | **0.4534** | **0.7764** |

The resulting deconvolved H-CHANNEL and DAB-CHANNEL images showed similar intensity levels in both cohorts (Figure 1). This procedure with same input vector was also used in the texture feature extraction CellProfiler ruleset.

- 1. **Resize**. After deconvolution the H-CHANNEL and DAB-CHANNEL are downsized by a factor of 2.
  2. **ApplyThreshold**. The DAB-CHANNEL was then used to segment pixels positive for Melan-A. To threat all images in both cohorts equally a manual threshold for separating high from low DAB intensity areas (threshold = 0.6).
  3. **Morph**. On the binary segmented DAB-CHANNEL image, a set of morphological operations are applied to group disjointed masked pixels into areas approximating the macroscopic tumor gland.
  4. **Resize**. The resulting image mask from the previous step is resized to the original size (Figure S1).

1. **Cell nuclei segmentation and feature extraction.** The CellProfiler ruleset used can be found in attached supplementary document 3
   1. **UnmixColors**. Stain separation (details given above).
   2. **Resize**. After deconvolution the H-CHANNEL, DAB-CHANNEL and Melan-A-MASK are downsized by a factor of 2.
   3. **Preprocessing prior to cell nucleus segmentation**. The key function of preprocessing is to improve the image in ways that increase the chances for success of the other processes. Typically deals with image analysis and processing techniques for enhancing contrast, removing noise, and isolating regions. During the preprocessing step, initially the H-CHANNEL image is sharpened and then morphological image operations are performed to highlight objects of interest (Figure 2B).
   4. **Segmentation of cell nucleus**. After preprocessing desired areas of the image (i.e. Melan-A or remaining) are selectively masked-in to segment cell nucleus. The cell nucleus segmentation identifies primary objects in the masked grayscale images containing bright objects on a dark background (Figure 2C-D). CellProfiler propose a modular three-step strategy to identify objects.
      - Initially the foreground (i.e. cell nuclei) is separated from the background. The threshold separating background using is automatically estimated for each image using a robust background global subtraction approach.
      - The edges of nuclei are identified, using thresholding if the object is a single, isolated nucleus, or if the object is actually two or more nuclei that touch each other. Nucleus objects that tend to have only one peak of brightness per object (e.g. objects that are brighter towards their interiors and dimmer towards their edges), counting each intensity peak as a separate object.
      - From the nucleus identified objects in previous step, those not meeting a minimum and maximum diameter size criteria are discarded. Such criteria were defined based on the optical properties of the digital image-capturing device. Based on the digital camera properties, two consecutively resolved pixels correspond to 0.25 **μm** in real object. The minimal and maximal diameters of a valid nucleus object were fixed to 10 pixels (2.5 **μm**) and 80 pixels (25 **μm**). All objects below and above these limits were discarded. This step identifies two set of nuclei, melanoma cell nucleus (Figure 2D) and the remaining stroma and normal epithelium cell nucleus (Figure 2C).
   5. **Measure morphological properties of cell nucleus**. Upon the measures melanoma and non-melanoma cell nucleus, several area and shape features are measures. A detailed description of these features is given in supplemental table 1.
   6. **Measure texture properties of cell nucleus**. The second set of features measured upon the two sets of nuclei objects is texture properties. This module quantifies intensity variation, radial distribution and granularity variations in the H-CHANNEL of the two sets of objects . A detailed description of these features is given in supplemental table 1.

Gabor, D. (1946). "Theory of communication. Part 1: The analysis of information." Electrical Engineers - Part III: Radio and Communication Engineering, Journal of the Institution of **93**(26): 429-441.

Haralick, R. M., S. R. Sternberg, et al. (1987). "Image analysis using mathematical morphology." IEEE Trans Pattern Anal Mach Intell **9**(4): 532-550.

Ruifrok, A. C. and D. A. Johnston (2001). "Quantification of histochemical staining by color deconvolution." Anal Quant Cytol Histol **23**(4): 291-299.

Serra, J. (1979). "[Biomedical image analysis by mathematical morphology (author's transl)]." Pathol Biol (Paris) **27**(4): 205-207.

Wahlby, C., I. M. Sintorn, et al. (2004). "Combining intensity, edge and shape information for 2D and 3D segmentation of cell nuclei in tissue sections." J Microsc **215**(Pt 1): 67-76.
